# Influence of Waste Glass Addition on the Fire Resistance, Microstructure and Mechanical Properties of Geopolymer Composites

**DOI:** 10.3390/ma16176011

**Published:** 2023-09-01

**Authors:** Celina Ziejewska, Agnieszka Grela, Dariusz Mierzwiński, Marek Hebda

**Affiliations:** 1 Faculty of Materials Engineering and Physics, Cracow University of Technology, Warszawska 24, 31-155 Kraków, Poland; celina.ziejewska@pk.edu.pl (C.Z.); dariusz.mierzwinski@pk.edu.pl (D.M.); 2 Faculty of Environmental Engineering and Energy, Cracow University of Technology, Warszawska 24, 31-155 Kraków, Poland; agnieszka.grela@pk.edu.pl

**Keywords:** fly ash, fire resistance, compressive strength, particle size, specific surface area

## Abstract

Nowadays, humanity has to face the problem of constantly increasing amounts of waste, which cause not only environmental pollution but also poses a critical danger to human health. Moreover, the growth of landfill sites involves high costs of establishment, development, and maintenance. Glass is one of the materials whose recycling ratio is still insufficient. Therefore, in the presented work, the influence of the particle size and share of waste glass on the consistency, morphology, specific surface area, water absorption, setting time, and mechanical properties of geopolymers was determined. Furthermore, for the first time, the fire resistance and final setting time of such geopolymer composites were presented in a wide range. Based on the obtained results, it was found that the geopolymer containing 20% unsorted waste glass obtained a final setting time that was 44% less than the sample not containing waste glass, 51.5 MPa of compressive strength (135.2% higher than the reference sample), and 13.5 MPa of residual compressive strength after the fire resistance test (164.7% more than the reference sample). Furthermore, it was found that the final setting time and the total pore volume closely depended on the additive’s share and particle size. In addition, the use of waste glass characterized by larger particle sizes led to higher strength and lower mass loss after exposure to high temperatures compared to the composite containing smaller ones. The results presented in this work allow not only for reducing the costs and negative impact on the environment associated with landfilling but also for developing a simple, low-cost method of producing a modern geopolymer composite with beneficial properties for the construction industry.

## 1. Introduction

During the past few decades, building has been one of the fastest-growing industries worldwide, and this trend is expected to continue, with global construction output reaching 42% growth in 2030 compared to 2020 [1]. Concrete is the most common building material around the world [2], and it consists primarily of Ordinary Portland Cement, a widespread type of cement that has been used in the construction sector for many years [3]. It is worth mentioning that global cement production has been still increasing; for instance, in 2000 it amounted to 1600 million tons, whereas in 2019 it reached 4100 million tons [4]. Although cement has many advantages such as great mechanical properties and durability, its production process has a meaningfully negative impact on the environment due to its high energy consumption and substantial emission of CO_2_ [5,6,7,8].

Glass is produced in around 100 million tons per annum, and it is a widespread material that is used worldwide in households, industries, laboratories, medicine, etc. Generally, it is believed to be an environmentally friendly material because it can be reused. However, the current global reuse rate for glass waste is only 26%, which results from the high cost of recycling [9]. Therefore, most of this waste is disposed of in landfills, causing environmental and economic problems. Glass is a non-biodegradable material, and it is estimated that millions of years are needed to decompose it naturally [10]. Scientists have been alarmed that global waste production is constantly growing and will double by 2050 and even triple by 2100 if the current trend continues, in comparison to the amounts observed in 2016 [11]. Due to that, the possibility of the application of waste in the construction industry has a growing interest [12]. It has been proven that waste glass can show pozzolanic reactivity, but only when its particle size is equal to or smaller than 100 μm [13]. Waste glass can play a different role in a matrix of cement concrete depending on its particle sizes, such as coarse aggregates (particle size smaller than 14 mm), fine aggregates (particle size below 4.75 mm), and binders (particle size below 0.6 mm) [14]. Furthermore, authors in their previous works used waste glass as a partial replacement for cement [15], or as a fine type of aggregate in mortar [16], fly ash [17], and metakaolin [18]. In addition, the properties of waste glass depend on several factors, such as its chemical composition, type and share of contaminants, the color of the cullet, and its origin [19]. The addition of glass can positively impact on mechanical strength [20], the capacity of radiation shielding [21], workability [22], density [23], and water absorption [24].

Fly ash is a by-product received most of all from power plants as a result of coal combustion [25]. Millions of tons of coal fly ash are produced annually all over the world, including, among others, 120–150 million tons from India [26], more than 550 million tons from China [27], and 145 million tons from Europe [28]. Although fly ash can be used primarily as an additive in concrete, its utilization rate is still very low; for instance, in Europe, in 2016, it amounted to only 20.1% [28]. The remainder of the materials are deposited in ever-expanding landfill areas.

Geopolymer is an excellent alternative to cemental materials due to the possibility of using industrial by-products and waste in their production (e.g., fly ash [29,30], gangue [31], slag [32], clay [33]), which is in line with the effective reduction of production costs. Moreover, the cement industry is responsible for approximately 6–8% of the total anthropogenic CO_2_ emission [34], whereas the geopolymer production process generates up to 5–6 times less quantity of greenhouse gas [35]. In general, geopolymers are inorganic polymers obtained by the reaction between aluminosilicate precursors and alkaline activators, which was invented by Joseph Davidovits in 1970 [36].

Despite the availability of many products reporting about the state of emergency, such as smoke detectors or temperature sensors, fires are still the key danger to people’s health and lives nowadays. The NSC (National Safety Council) organization estimated that fire brigades in the United States react, on average, every 23 s to a fire occurrence, and every 3 h and 8 min, approximately, one person dies because of it. Therefore, fire resistance is one of the most important features of building materials, due to the necessity to ensure the stability of the construction during the fire [37]. Concrete manufactured using Ordinary Portland Cement shows significant deterioration of properties at temperatures above 400 °C because of the decomposition of portlandite. Moreover, in the range of 300–450 °C, spalling of such type of concrete is noted [38]. In contrast, geopolymers have good thermal stability and do not emit noxious vapor pending the fire, which is particularly important with regard to user safety [38]. Therefore, they can be applied as thermal insulation, as well as fire protection [3,39]. It was proven that even a small amount of geopolymer incorporated into a concrete matrix has a positive impact on fire resistance [40].

It was found that waste glass could have a beneficial impact on fire resistance due to the transition from the solid to liquid state, which is directly related to the melting point of waste glass. At a proportionately high temperature, waste glass can have the ability to fill pores, and can also fill microcracks that occur as a result of high temperatures [41]. Until now, many authors explored the fire resistance of concrete materials and cement mortars [42,43]. For instance, Chen et al. [44] examined the influence of the size of glass cullet on cementitious composites after higher temperature exposure using four various fractions of particles of the following sizes: less than 0.6 mm, 0.6–1.18 mm, 1.18–2.36 mm, and 2.36–4.75 mm. The authors concluded that only samples containing two of the smallest fractions of particles improved the residual compressive strength after exposure to a temperature of 600 °C or higher, compared to the reference sample produced without adding the cullet. Moreover, they highlighted that the most positive effect was obtained by applying particles with sizes below 0.6 mm. However, in the case of geopolymers with the addition of waste glass, investigations about their fire resistance are very limited. Tahwia et al. [45] investigated the effect of high temperature on the properties of geopolymer concrete made with the addition of waste glass and ceramic after 56 days of curing. They incorporated waste glass particles into a matrix in the amount of 7.5 to 22.5% by volume and later subjected the samples to high temperatures of 200–800 °C for 1.5 h. The researchers noticed that the compressive strength of all the samples produced, even the control samples, was higher after exposure to 200 °C due to the further course of the geopolymerization process. However, after exposing the samples to a temperature of 800 °C, the authors noted the beneficial effect of glass waste, which resulted in an increase in residual compressive strength compared to the control mixture containing ceramic waste. Furthermore, Jiang et al. [46] explored fly ash-based geopolymers with the addition of waste glass (D_50_ = 18.21 μm) before and after exposure to 800 °C, 1000 °C, and 1200 °C. They concluded that the optimal level of incorporation of waste glass was 20% and after achieving the melting temperature of the glass, it can fill pores existing in the geopolymer matrix. Yu et al. [47] investigated the influence on the fire resistance of two types of waste glass with sizes of 4.9–10 mm and 4.9–16 mm, which were used as a coarse aggregate in the concrete columns. At the beginning of the test, samples were subjected to compressive loading, and then they were placed in the furnace and heated by up to 800 °C. The most positive effect was noted for samples containing 13% waste glass, and those samples exhibited longer endurance during the fire than concrete ones. Turkey et al. [47] tested lightweight geopolymer concrete with the addition of glass powder, which was ground to achieve particles of 10–30 µm. They found that after heating at 200 °C the mechanical strength was higher, and it decreased at 400 °C afterward. However, the most significant decrease was observed at 800 °C, which was caused by the loss of water from the structure of materials. Moreover, Chindaprasirt et al. [48] confirmed the profitable effect of the incorporation of fine auto glass on the fire-resistance performance of fly ash-based geopolymers.

Until now, the effect of incorporating waste glass with various particle sizes and shares on geopolymer fire resistance remains unknown. Therefore, the main objective of this work is to fill the existing research gap. The consistency of fresh geopolymer mortars was examined. The thermal effects occurring during the geopolymerization process were investigated using negative temperature coefficient thermistors. Mineralogical and mechanical analysis, microscopic observation, water absorption tests, density, and specific surface area of composites after curing depending on particle size and share of waste glass addition were carried out. Finally, a fire resistance test was performed, and then the loss mass, residual compressive strength, and chemical compositions of geopolymers were determined.

## 2. Materials and Methods

### 2.1. Materials

**Figure 1:**
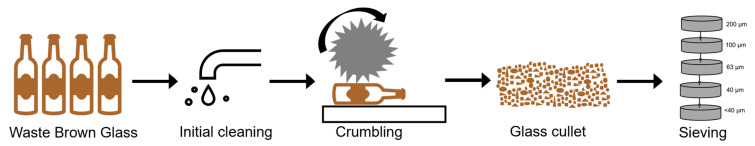
Preparation of waste glass for the experiments.

Fly ash from Skawina Power Plant was used as a primary material for the geopolymers synthesis. A commonly available quartz river sand (Swiętochłowice, Poland) was applied as an aggregate in the geopolymer matrix. Waste cullet glass of brown bottles after initial cleaning and crushing, used as a partial replacement of fly ash in geopolymer composites, was delivered by Grabowski Import Export Company (Sędziszowa, Poland). The glass cullet was sieved through a set of sieves with mesh sizes of 200 μm, 100 μm, 63 μm, and 40 μm to determine the influence of various ranges of waste glass particle size on the properties of the composites. As a result, there were six various ranges of particle sizes, such as 0.1–1200 μm (unsorted waste glass), 200–1200 μm, 100–250 μm, 63–120 μm, and 40–63 μm, 0.1–40 μm. The cullet was not subjected to additional cleaning before the application due to economic and environmental reasons, including reducing the cost of geopolymers production, as well as water and electricity savings. The schematic of the waste glass preparation process is shown in Figure 1.

Furthermore, Table 1 presents the particle size distribution of raw materials, such as fly ash, sand, and various factions of waste glass.

The alkaline activator consisted of an 8 M sodium hydroxide (NaOH) solution and an aqueous solution of sodium silicate mixed in the proportion 1:2.5. Sodium hydroxide (NaOH) in flake form, acquired from KRAKCHEMIA S.A. (Kraków, Poland), and tap water were used to obtain the NaOH solution, whereas an aqueous solution of sodium silicate (R-145) was bought from Chemi Kam Sp. z o.o. (Będzin, Poland).

### 2.2. Geopolymer Manufacturing

Fly ash, waste glass, and sand were stirred in a planetary mixer (GEOLAB, Warsaw, Poland) for 2 min, and then an alkali activator was slowly added to the mixture. The quantity of sand was equal in all produced samples, and it amounted to 10% by weight. This was decided on the basis of the literature review in order to increase the durability property of geopolymers, as well as reduce the shrinkage and porosity of manufactured samples [50]. The waste glass as a replacement for fly ash was added at a weight percent of 10%, 20%, and 30% for each fraction of particle size. Based on the literature information, it can be concluded that the optimal share of the addition of waste glass to building materials is usually between 10–30% [51]. The liquid-to-solid ratio was established based on the tested workability, and it was 0.4. Well-mixed homogeneous blends were cast into wood molds of appropriate dimensions, vibrated to dispose of inner air bubbles, and covered by stretch wrap. After drying, they were removed from the molds and then cured under laboratory conditions (at a temperature of 22 °C ± 3 °C and a humidity of 30% to 50%). Samples were designated in accordance with the applied share and the particle size of various fractions of waste glass in geopolymer blends (Table 2).

### 2.3. Characterization of Geopolymer Samples

**Figure 2:**
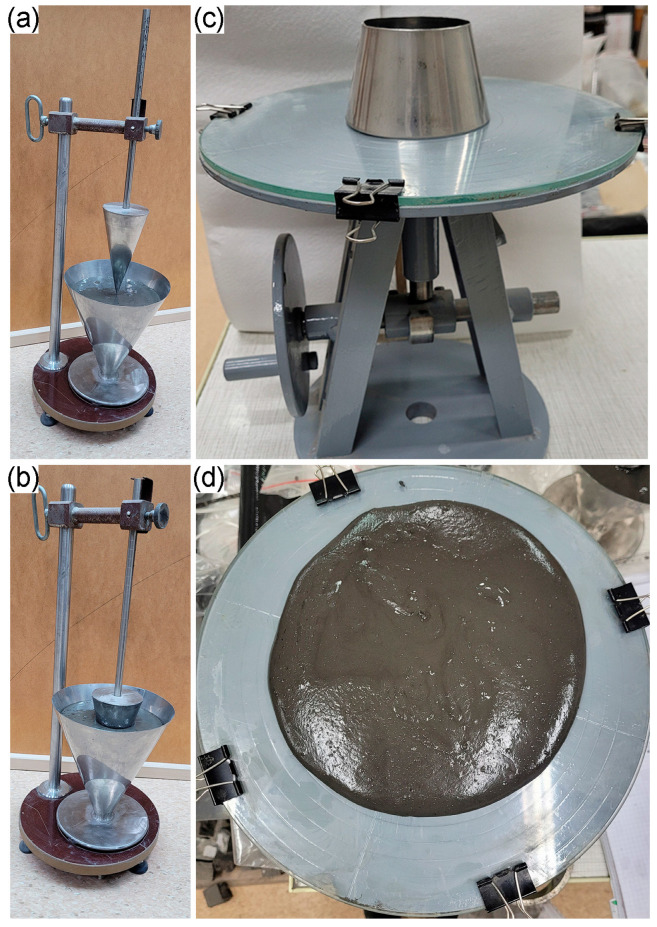
Consistency of geopolymers mortars determined by (**a**,**b**) the Novikov cone method and (**c**,**d**) the flow table test.

The consistencies of fresh geopolymer mortars were examined using two methods, the Novikov cone subsidence and the flow table test. The procedure for determining consistency is shown in Figure 2. Determination of consistency by the flow table was performed according to standard PN-EN 1015-3:1999/A2:2006 [52], whereas the Novikov cone test was acquired in accordance with PN-85/B-04500 standard [53].

The chemical composition of raw materials and geopolymers was determined using an EDX-7200 Energy Dispersive X-ray Fluorescence Spectrometer (Shimadzu Corporation, Kioto, Japan).

The nitrogen adsorption–desorption isotherms were determined using Autosorb—iQ/MP Quantachrome gas sorption analyzer (Anton Paar company, Graz, Austria). The test samples had been outgassed at 300 °C for 24 h prior to measurement to remove impurities from solid surfaces. The Brunauer–Emmett–Teller (BET) method was applied to calculate the specific surface area of geopolymers.

The density of geopolymer composites was calculated by dividing the samples’ mass by volume (geometric method). The mass was measured by using a Radwag PS 200/2000R2 (RADWAG Wagi Elektroniczne, Radom, Poland) precision balance. The final result was the average of the measurements of the 3 samples.

The mineralogical composition of geopolymers was determined by X-ray diffraction using PANalytical Aeris Diffractometer (Malvern Panalytical, Almelo, The Netherlands, Cu Kα radiation) with the step size of 0.003° (2θ), a time per step of 340 s, and the 2θ angular range of 10–100°.

The mechanical properties of the samples were investigated using the concrete press MATEST 3000 kN (Matest, Treviolo, Italy). The compressive strengths were tested in accordance with the PN-EN 12390-3:2019 standard using at least three 50 mm cubical samples of each geopolymer composition after 56 days of curing [54].

The microstructure of geopolymer composites was analyzed using a JEOL JSN5510LV model Scanning Electron Microscope. Before the investigation, the surface of the geopolymers was coated with a thin gold layer by using a JOEL JEE-4X vacuum evaporator (JEOL, Tokyo, Japan).

The fire resistance of geopolymers was determined in accordance with the PN-EN ISO 1182:2020 standard [55]. The samples were placed inside an electric furnace at a temperature of 750 °C. After the isothermal holding of the samples for 30 min, they were cooled together with the furnace to room temperature. Then, the mass loss of specimens was determined according to the following equation:

$$Loss\;of\;mass,\;\%=100\times \left[1-\frac{mass\;after\;experiment}{mass\;before\;experiment}\right]$$

Moreover, the residual compressive strength of samples after the fire resistance test was examined and the result was the arithmetic average of a minimum 3 specimens for each type of geopolymer.

The water absorption test of geopolymers was performed in compliance with PN-88/B-06250 ‘ordinary concrete’ standard. For performing this, three cubic samples for each type of geopolymer were immersed in water to half of their height. After 24 h, water was added in the proper amount to obtain a 10 mm higher water level than the height of samples, and this level was held until the end of saturation. Samples were removed from the water, and after wiping the surface, they were weighed every 24 h. The investigation was ended when two consecutive weighings showed a lack of increase in weight. Completely saturated samples were placed in a dryer until a constant mass was obtained.

The following dependence was applied to determine the water absorption:

$$n_{w}=\frac{G_{2}-G_{1}}{G_{1}}\cdot 100\;[\%]$$

where *G_1_* and *G_2_* were the average mass of dry specimens and the average mass of the samples replete with water, respectively.

To explore the geopolymerization process depending on the waste glass addition, the fresh geopolymer mortars, and the geopolymers after 48 h of curing, were placed in a laboratory dryer at 75 °C, and distinctive effects were determined using a dedicated negative temperature coefficient system, as described in our previous work [56]. Samples were selected to test based on the most significant differences in the size of the waste cullet (20_0.1–40 and 20_200–1200) and its content in the geopolymer matrix (20_0.1–1200 and 30_0.1–1200). The reference sample contained no added glass. In addition, in order to determine the effect of sand addition on the geopolymerization process, a fly ash-only sample was analyzed.

## 3. Results and Discussion

The chemical compositions of fly ash, sand, and waste glass are presented in Table 3. Based on the obtained results, coal fly ash used in the experiment mainly consisted of silica, aluminium oxide, and iron oxide. The residual carbon content of fly ash expressed as LOI (the loss of ignition) reached the value of 3.284 [57]. Therefore, it can be classified as class F according to the ASTM C618-95 standard [58]. An extensive study of raw materials was provided in our previous work [49].

**Figure 3:**
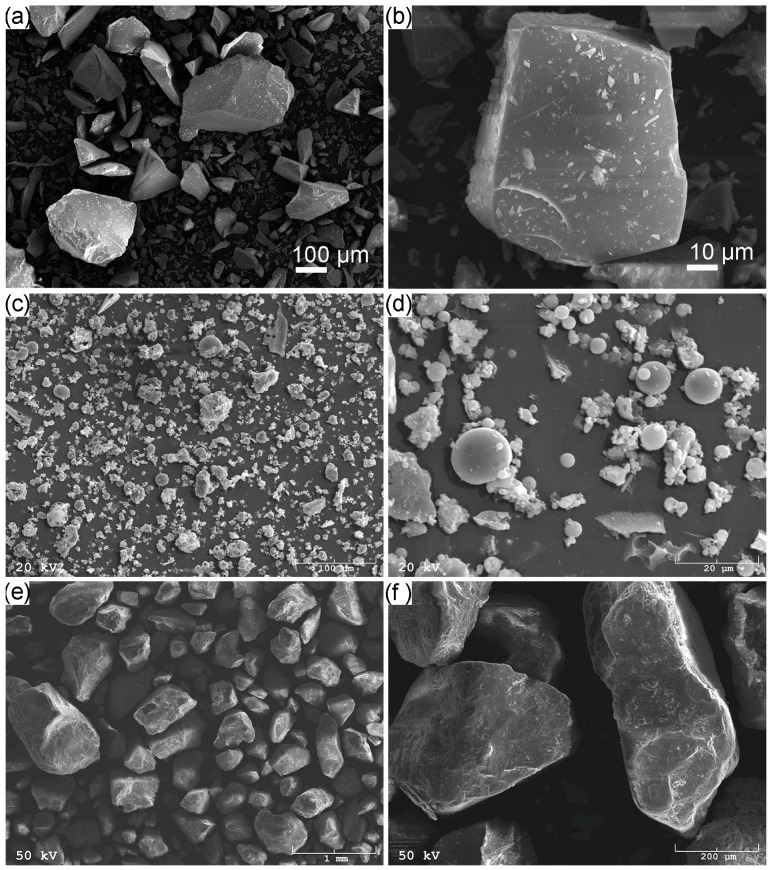
SEM micrographs of (**a**,**b**) waste glass, (**c**,**d**) fly ash, and (**e**,**f**) sand.

Figure 3 presents the morphology of (a,b), waste glass in the delivery condition, (b,c) fly ash, and (c,d) sand. The waste glass consisted of particles characterized by irregular shapes, diverse sizes, and sharp edges. The river sand had irregular angular structures with hollows, flutes, and protruding sections. The fly ash that was used as a base material in the experiment contained irregular and spherical particles, however, with a predominance of spherical ones, facilitating the geopolymerization process [59].

**Figure 4:**
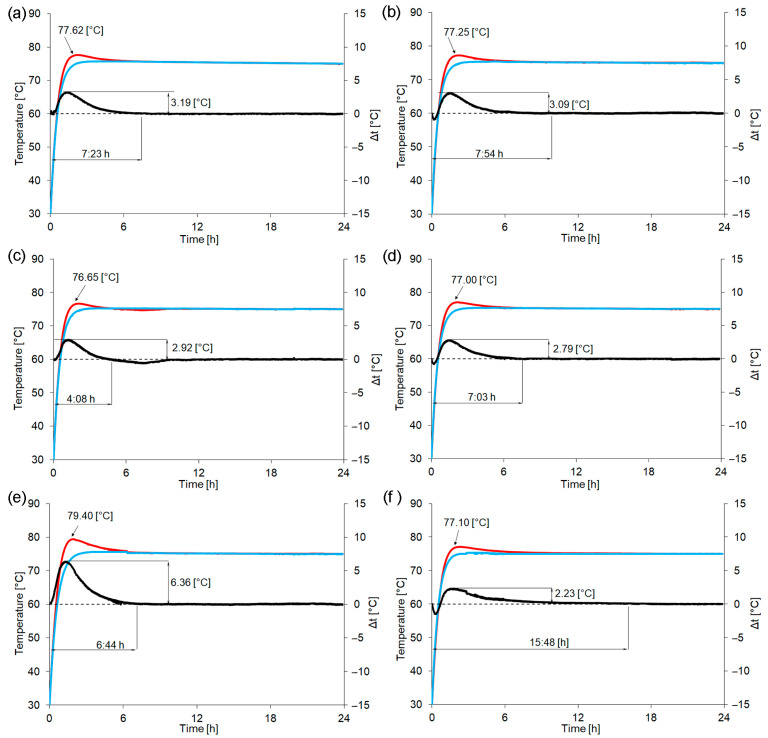
Thermal effects of (**a**) REF, (**b**) REF without sand, (**c**) 20_0.1–1200, (**d**) 20_200–1200, (**e**) 20_0.1–40, and (**f**) 20_0.1–1200 registered using a negative temperature coefficient thermistor device for fresh geopolymer mortars during the geopolymerization process (red curve), after 48 h of curing (blue curve), and the difference between these two curves (black curve).

Figure 4 demonstrates the obtained results, and three curves were presented on the graph for each sample, representing thermal effects that took place pending the geopolymerization process of fresh geopolymer mortar (red curve), those that took place after 48 h of curing (blue curve), and the difference between these two curves (black curve).

Furthermore, Table 4 summarizes determined characteristic values, such as the maximum gained temperature during the geopolymerization process of fresh geopolymer mortar and the corresponding time, the final setting time, the energy of exothermic reaction, and the temperature difference between the maximum values of thermal effects recorded during the geopolymerization process of fresh geopolymer mortars and after 48 h of curing.

In the comparison of materials containing various particle sizes of waste glass addition, it has been seen that applications of the smallest particles resulted in the following: (1) the highest reaction energy and the highest recorded temperature, (2) the largest difference between the maximum recorded values of thermal effects, and (3) the maximum thermal effect that was recorded earlier than for the other samples. These effects can be explained by the fact that smaller particles are characterized by larger specific surface areas, and, therefore, the larger area is accessible to the alkaline activator during the geopolymerization process [60]. The presence of exothermic effects results from the dissolution of the solid raw material in the alkaline activator and thus the formation of new material phases. Moreover, it is clearly visible that the energy of the exothermic reaction and achieved maximum temperature depended on the size of the waste glass and were the highest in the case of the sample containing the smallest particles (20_0.1–40), which testified to the highest soluble of these raw materials during the geopolymerization process [61]. During the geopolymerization process, the amount of released heat is relatively small, indicating that occurring reactions are rather thermally stable [56].

In general, it was found that the addition of 20% waste glass decreased the final setting time regardless of the used fractions. Basically, the incorporation of starting materials including a higher content of calcium may impact the reduction of the setting time and, simultaneously, the increase of mechanical strength, and this was proven by the presented results [62]. Noteworthily, the 20_0.1–1200 sample, containing unsorted waste cullet, had the shortest final setting time, and, more specifically, it constituted the following part of the final setting time of other samples: 52% of REF without sand, 56% of REF, 59% of 20_200–1200, 61% of 20_0.1–40, and 8% of 30_0.1–1200. However, the elevation of the addition of unsorted waste glass to 30% (30_0.1–1200) caused a different effect, namely, the final setting time was the most extended and was more than twice as long as for the REF sample. The shorter final setting time of geopolymer could be beneficial in terms of future industrial applications. Moreover, considering the effect of sand on the geopolymerization process, it was found that the use of 10% sand resulted in a decrease in the energy released during the exothermic reaction and a shortening of the final setting time.

**Figure 5:**
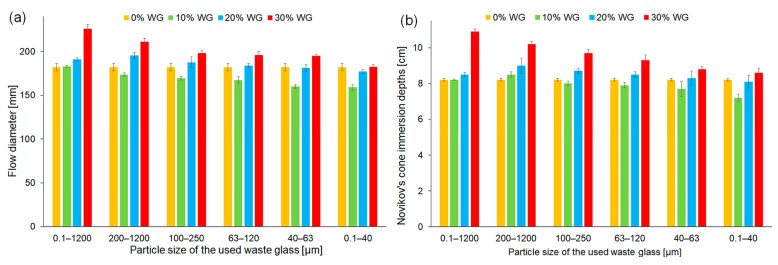
The consistency of fresh geopolymer mortars depending on the share and the fraction of waste glass particle size determined by (**a**) the flow table test and (**b**) the Novikov cone method.

As it is commonly known, consistency is defined as the ability of a fresh mixture to flow. Its properties depend on the type of raw materials used, particle size, number of components, and liquid-to-solid ratio [63]. Figure 5 shows the results of the consistency assessment of fresh geopolymer mortars measured by the flow table test and Novikov cone methods. The reference mortar, without the addition of waste glass, achieved values of 182 mm and 8.2 cm by using the Novikov cone and flow table methods, respectively. Moreover, on the basis of the recorded results, it can be seen that all of the fresh geopolymer mortars containing up to 20% of waste glass had plastic consistency. This effect was regardless of the particle size. However, the addition of 30% of waste cullet with particle sizes ranging between 0.1 and 1200 µm or 200 and 1200 µm resulted in a change in the consistency of the mortar to liquid/thin. The explanation of this phenomenon is related to the fact that waste glass particles with larger dimensions do not have the ability to absorb as much alkaline solution as fly ash. In contrast, the use of cullet in the same amount (30% by weight) but with a smaller particle diameter determined the formation of a mixture with a plastic consistency. It should be noted that decreasing the particle size of waste glass addition reduces the flow diameter and Novikov’s cone immersion depth of fresh geopolymer mortars. This effect can be explained by the increase of the specific surface area of smaller particle waste glass, which absorbed a larger quantity of alkaline solution. Other authors noticed a similar phenomenon. The results clearly indicate that the flowability of composite with the addition of glass depends on a few factors, including the type, size, and roughness of the incorporated addition [64,65].

**Figure 6:**
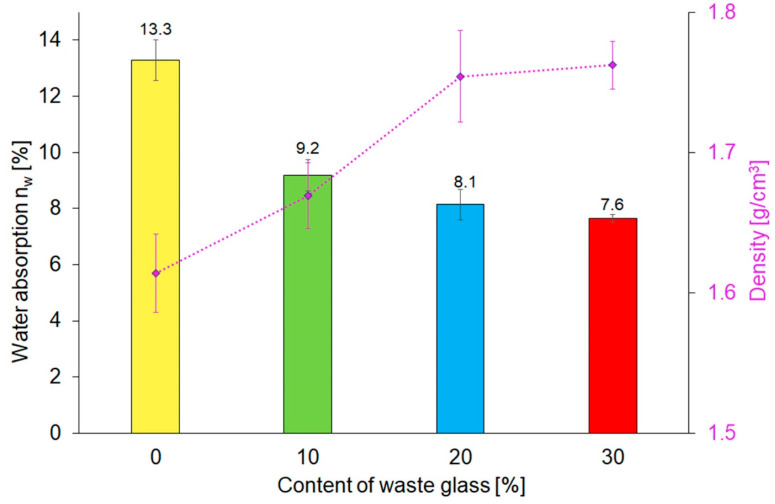
Water absorption and density of geopolymers depending on the composition of the samples.

Water absorption is an important feature of construction materials, which shows the resistance of concrete materials to sulphate, chloride ions, carbonation, and freeze-thaw cycles [66]. Water absorption is strongly connected to pore size and volume in the investigated materials [67]. Figure 6 presents the results of the water absorption and density test of reference geopolymers and composites containing unsorted waste glass, corresponding to the particle size of 0.1–1200 µm. There was a strong dependence between the share of waste glass in geopolymers and their water absorption. As predicted, the water absorption decreases with the increase of waste glass share in geopolymers. The reason for this is the water absorption of waste glass, which is close to zero [68,69]. Furthermore, the addition of waste glass to the geopolymer matrix resulted in higher density as compared to the reference material.

**Figure 7:**
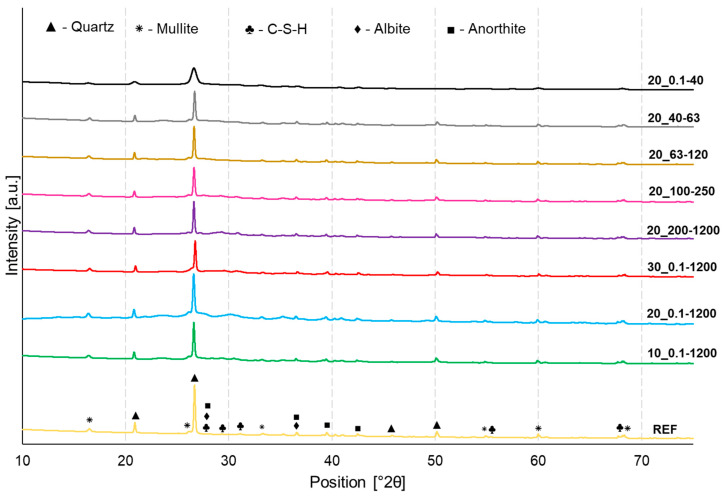
XRD analysis of reference samples and selected geopolymers depending on the share and the fraction of waste glass particle size.

In order to assess the influence of waste glass on the mineralogical composition of geopolymers containing different shares and sizes of the fraction of the waste glass addition, their XRD patterns were compared (Figure 7). The qualitative analysis revealed the following phases: quartz (SiO_2_, ref. code: 01-075-8320), mullite (Al_6_Si_2_O_13_, 01-082-1237), C-S-H as Rosenhanite (Ca_3_ (Si_3_O_8_(OH)_2_, ref. code: 00-029-0378), albite (NaAlSi_3_O_8_, ref. code: 01-071-1150), and anorthite (CaAl_2_Si_2_O_8_, ref. code: 04-013-2357). In general, the peak locations of all investigated samples had almost no difference, pointing out that they consisted of the same phases. However, the XRD peaks of samples manufactured using waste glass addition had lower intensity as compared to the reference material. Similarly, the smaller the particle size, the lower the intensity of the peaks, which is especially evident for the sample containing the smallest particle size (20_0.1–40). This effect can be attributed to the decline in the degree of crystallinity. The appearance of the hump on the diffractogram at position 15–35° 2θ indicates the presence of an amorphous phase, which was identified as a C-S-H compound. Moreover, sharp diffraction peaks observed in particular positions on the diagram as a result of the use of X-rays were assigned to crystalline phases [70]. Albite and anorthite belong to plagioclase feldspar minerals, and due to their high stability, they can serve as a filler or reinforcing material [71].

**Figure 8:**
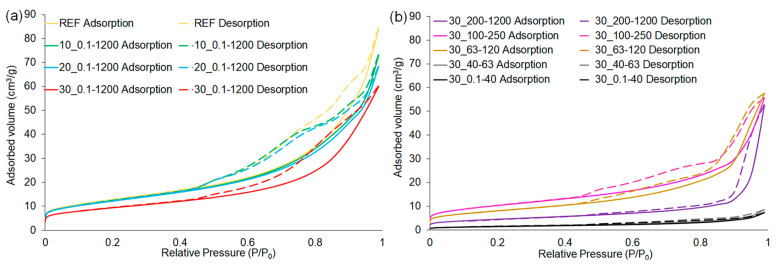
Nitrogen adsorption–desorption isotherms of geopolymers depending on: (**a**) the share and (**b**) the fraction of waste glass particle size.

The nitrogen adsorption–desorption isotherms of samples containing various shares and particle sizes of waste glass are presented in Figure 8a,b, respectively. According to the IUPAC (International Union of Pure and Applied Chemistry) classification, all obtained isotherms can be categorized as type IV isotherms with H3 hysteresis loops [72]. In this type of isotherm, a hysteresis loop occurs as a result of the filling or evacuating of mesopores (pores with a diameter of 2–50 nm) via capillary condensation [73,74].

The specific surface areas of geopolymers calculated by the single-point and multi-point Brunauer–Emmett–Teller (BET) methods, pore volume, and pore size are shown in Table 5. It can be noticed that the specific surface area of the 30_0.1–1200 sample achieved 33.26 m^2^ g^−1^; therefore, it decreased by almost 27% compared to the reference sample, taking into account the result of the multi-point BET method. Furthermore, the smallest specific surface area of 5.35 m^2^g^−1^ was obtained for the sample with the 30% addition of the smallest particle size (30_0.1–40), representing a decrease of around 88% and 84% in comparison with the sample without the additive and with 30% of waste glass characterized by the largest particle size, respectively. Geopolymers including the largest range of waste glass particle size (0.1–1200 μm) showed a decrease in specific surface area with an increasing share of addition. Based on the recorded results, it was found that the specific surface of the samples decreased with the use of smaller particle sizes of glass waste, as well as with the share increase of glass addition to the composite.

The application of 30% of waste glass with particle sizes ranging from 0.1 to 1200 μm resulted in a decrease of 29% of the total pore volume as compared to the reference specimen. Furthermore, the total pore volume in the sample containing 30% waste characterized by the smallest particle sizes (30_0.1–40) was over 11 times lower than the reference material, indicating that this factor closely depended on the share and particle size of the additive. The obtained results also showed a certain, but not so clear, correlation in terms of the pore diameter of geopolymers, i.e., the smaller particle size and higher share of the waste glass, the lower the diameter of pores were.

The findings were consistent with the density and water absorption of geopolymer composites. The incorporation of waste glass caused a decrease in water absorption, a reduction in pore size and volume, a slight increase in density, and a cutting of specific surface area simultaneously. These results show that the addition of waste glass reduces the porosity of the fly ash-based geopolymer matrix, causing the formation of denser structures.

**Figure 9:**
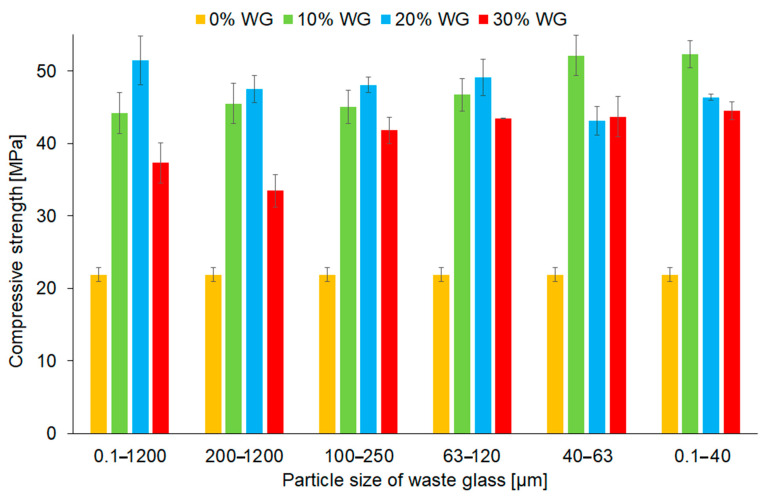
The compressive strength of geopolymers depending on the share and the fraction of waste glass particle size.

The compression strength of geopolymers depending on the share and particle size of the waste glass addition was demonstrated in Figure 9. The compressive strength of the geopolymers increased after the incorporation of waste glass into the matrix by 53% to 137%, depending on the composition of the samples. Similar results were obtained by Jiang et al. [46], who explained that glass contains SiO_2_ and Al_2_O_3_, which can react and form aluminosilicate hydrates, which have a positive influence on compressive strength.

It is evident that the optimal level of the waste glass changed depending on the particle size of the addition, and it amounted to 20% for samples containing the following fractions: 0.1–1200 μm, 200–1200 μm, 100–250 μm, and 63–120 μm. However, in the case of using fractions with smaller particles, 63–40 and 0.1–40 μm, the optimal level was achieved with a 10% addition of waste glass. This phenomenon is related to the pozzolanic activity of waste glass, which depends mainly on the chemical composition and particle size. The crumbling of waste glass could lead to the enhancement of its pozzolanic reactivity and could react in the geopolymerization process afterward [23]. However, when the share of waste glass amounted to 30%, the Si/Al ratio was higher, resulting in the formation of low-crosslinked aluminosilicates, and therefore the deterioration of mechanical properties [75]. It is worth mentioning that all geopolymer composites, even with 30% of waste glass, had higher results than the reference material.

Fly ash showed a mean particle size of 17.3 ± 2.5 µm, whereas the fraction of waste glass containing the smallest particle size (0.1–40 µm) had a mean particle size of 22.0 ± 0.3 µm. On the other hand, the true density of fly ash and waste glass was 2.288 ± 0.001 g cm^−3^ and 2.507 ± 0.001 g cm^−3^, respectively. In general, using smaller particle sizes of starting materials is beneficial due to the higher available specific surface area and therefore higher reactivity and more efficient course of geopolymerization process [57]. Even though fly ash had a smaller particle size, the replacement of it by waste glass in geopolymers was beneficial in terms of compressive strength, regardless of the used fraction of waste glass. It is evident that the main factor affecting the strength properties of samples was the ability to dissolve silica-rich waste glass particles. Furthermore, waste glass contained a higher amount of CaO (11.671%) than fly ash (4.698%), resulting in the development of additional calcium silicate hydrate and therefore structures that are characterized by higher density and strength, and this was also noticed in previous work [76]. Regarding the particle size of waste glass, many earlier reports indicated that using the finer particles resulted in a higher strength of the final material due to their higher pozzolanic reactivity [77]. A similar tendency is also visible in the presented study, showing that the most positive effect of the incorporation of waste glass on the compressive strength was achieved for samples containing the smallest particle size (0.1–40 μm). However, it should be noted that surprisingly high compressive strength was obtained using unsorted waste glass, consisting of the widest size range of particles.

**Figure 10:**
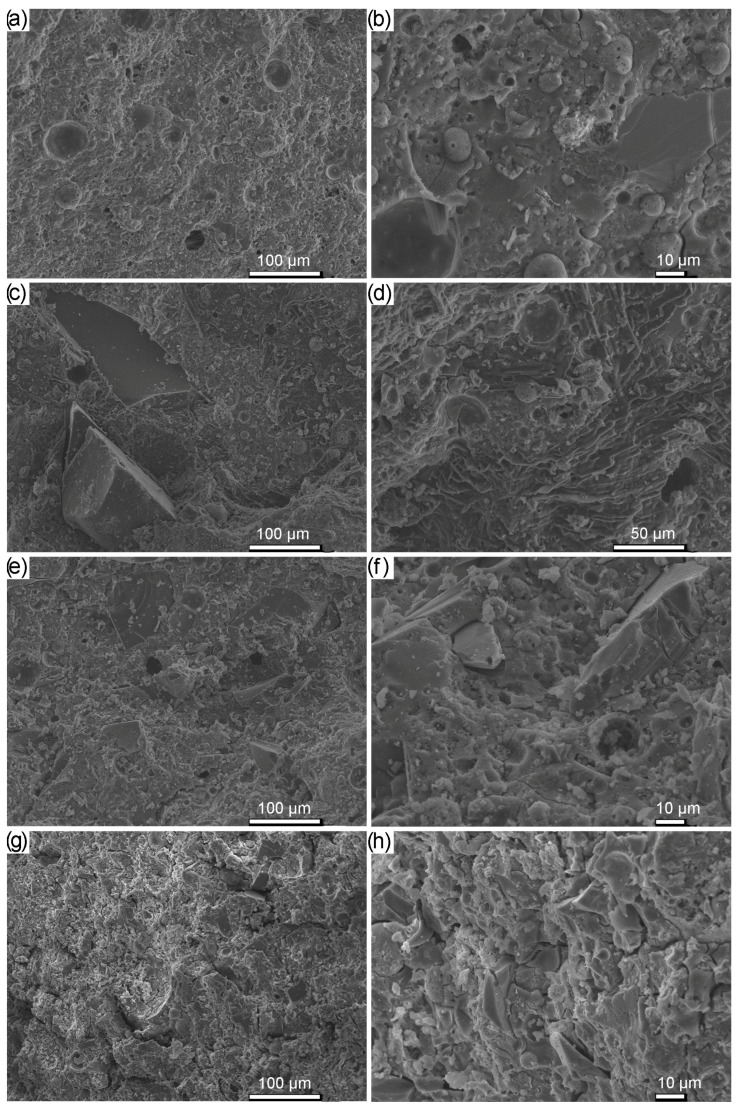
SEM images of geopolymers after the compressive strength test depending on the share and the fraction of the waste glass particle size: (**a**,**b**) REF, (**c**,**d**) 30_200–1200, (**e**,**f**) 30_63–120, (**g**,**h**) 30_0.1–40.

The morphology of the reference material and samples with 30% share and various sizes of the waste glass is presented in Figure 10. Because the samples were observed after the compressive strength test, cracks can be seen on their surface. A greater number of pores in the matrix of reference samples was clearly visible, compared to composites with the addition of glass waste. This observation was consistent with the geopolymer density results presented earlier. Moreover, unreacted particles of fly ash in small amounts have occurred in the geopolymer matrix, which adversely affects the compressive strength [78]. On the other hand, it is well-known that during the geopolymerization process, not all of the components are converted into geopolymers.

**Figure 11:**
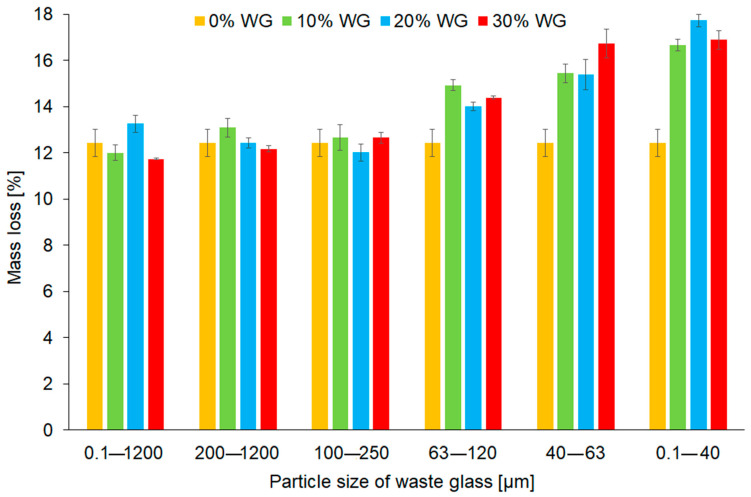
Mass loss of the geopolymers depending on the share and the fraction of waste glass particle size after the fire resistance test.

Figure 11 presents the mass loss of the geopolymer composites after the fire resistance test. The mass loss ranged from 11.7% (30_0.1–1200 sample) to 17.7% (20_0.1–40 sample). By comparing the mass loss of geopolymers, it can be concluded that reducing the particle size of addition caused a more significant weight loss of the samples after the fire resistance test. The explanation for this can be the amount of absorbed water inside the waste glass particles. As mentioned before, smaller particles of waste glass have a higher specific surface area and can absorb more water during the manufacturing process than coarse ones. The mass loss of geopolymers occurs at high temperatures mainly due to the effects of evaporation of adsorbed water, as well as the desquamation and chips of materials. However, depending on the temperature, various changes in the material were observed. In the beginning, at temperatures up to 400 °C, the fly ash-based geopolymers showed an improvement in compressive strength due to the reaction of unreacted particles of waste glass and the shrinkage of the pores. In higher temperatures, further changes took place, such as the transformation of iron compounds (400–800 °C), the formation of new pores with different sizes (400–800 °C), and the removal of the porosity during the sintering process (600–1000 °C), cracking of samples (600–800 °C), and shrinkage (200–1000 °C) [38,79]. However, regardless of the share of the added glass waste and its particle size fraction, it was found that all investigated composites can be categorized as materials class A1_fl_ according to the fire classification PN-EN ISO 1182:2020 standard.

**Figure 12:**
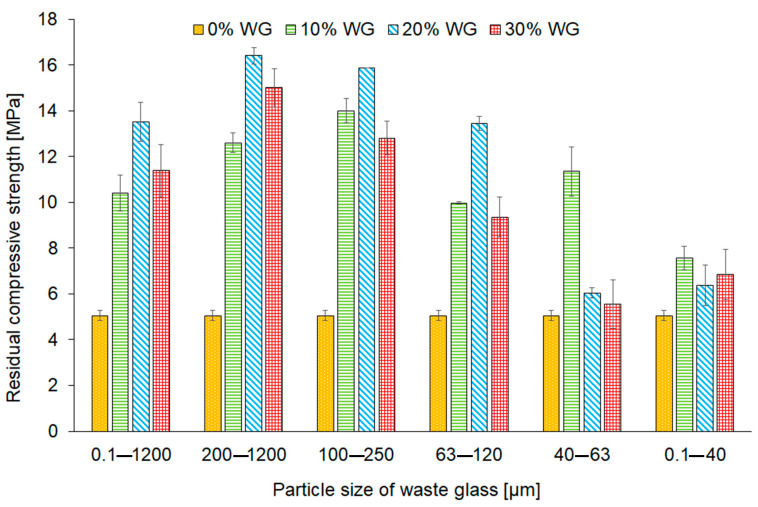
The residual compressive strength of geopolymers depending on the share and the fraction of waste glass particle size.

The influence of the high temperature on residual compressive strength changes of the samples after the fire resistance test was also investigated (Figure 12). The recorded results showed that, regardless of the size of the particle fraction and their share in the composite, the addition of glass waste had a beneficial effect on the residual compressive strength compared to the reference material. This effect was due to the introduction of an additional share of aluminium and silica into the material composition [80]. Bisht et al. [81] investigated the compressive strength of concrete mixes with the addition of waste glass after exposure to temperatures ranging from 200 °C to 800 °C. They noticed the beneficial impact of waste glass incorporation after exposure to 400 °C compared to the reference samples made without the glass addition.

It was noticed from the presented results (Figure 12) that the application of waste glass characterized by larger particle sizes led to reaching a higher strength than in the case of incorporating smaller particles. Similar observations regarding the influence of waste glass particle size on the compressive strength after exposure to high temperatures were made by Mao et al. [82], who investigated clay bricks with the addition of waste glass powder after heating at 950 °C for 3 h. They concluded that incorporating larger particle sizes of waste glass into brick resulted in higher compressive strength due to the formation of albite or glass–ceramic compounds. However, it should be emphasized that they investigated only the influence of waste glass with average sizes of 250, 100, 35, and 10 μm.

Interestingly, comparing the results of compressive strength before (Figure 9) and after (Figure 12) the fire resistance test it was found that the optimal share of waste glass for each fraction of particle size was the same. Considering the waste glass with particle sizes within the ranges of 0.1–1200 μm (unsorted cullet), 200–1200 μm, 100–250 μm, and 63–120 μm it can be seen that 20% of the additive resulted in achieving the best outcomes. Nevertheless, geopolymer composites with the addition of smaller particle sizes of the additive (fractions of 40–63 μm and 0.1–40 μm) achieved the best performance by applying 10% of waste.

Moreover, on the basis of the results, it was observed that the application of waste containing fractions with the highest particles (200–1200 μm) and (0.1–1200 μm) caused the increase in residual compressive strength by about 221.6% and 164.7% as compared to the reference geopolymer. Considering future industrial applications, it should be noted that the sieving and dispensing of waste glass are associated with high costs and there is a necessity to use additional technological solutions. Therefore, the results obtained for geopolymers with the additive of unsorted waste glass are the most promising.

The residual compressive strength of the geopolymers constituted 26.2% and 23.3% of compressive strength before the fire resistance test in the case of 20_0.1–1200 and the reference samples, respectively. It could be clarified that waste glass with smaller particle sizes shows higher pozzolanic reactivity and could react in the geopolymerization process. After being heated to 750 °C, the glass waste particles could change their state from solid to liquid due to the exceeded melting threshold of waste glass (below 700 °C) and hence could fill pores inside the samples [48,83].

The chemical composition of the selected geopolymers depending on share and particle size fractions of waste glass before and after the fire resistance test is shown in Table 6.

All samples consisted of three main components, SiO_2_, Na_2_O, and Al_2_O_3_, either before or after the fire resistance test. The total share of these main chemical compounds ranged from 81.881% (REF sample before the test) to 86.430% (30_200 sample before exposure to high temperature).

Before the exposure of geopolymers to high temperatures, the SiO_2_/Al_2_O_3_ ratio amounted to 3.45, 4.012, and 4.635 in REF, 30_200–1200, and 30_0.1–40 samples, respectively. Dehghani et al. [84] discovered that increasing the SiO_2_/Al_2_O_3_ ratio caused a decrease in the BET-specific surface area in fly ash-based geopolymers, and this dependency is also clearly noticeable in the presented study. Furthermore, Klima et al. [38] indicated that in the temperature range of 600–800 °C, oxidation of iron oxides contained in unreacted particles of fly ash takes place, which was also proved in this work. The XRF analysis showed that the Fe_2_O_3_ share in geopolymers after the fire exposure decreased by 19.3%, 11.7%, and 35.9% for samples designated as REF, 30_200–1200, and 30_0.1–40, respectively, as compared to the amount of this compound before treatment. In addition, it was noted that after the fire resistance test, SiO_2_ content in geopolymers increased by 21.9%, 16.4%, and 15.5%, whereas the content of Na_2_O decreased by 25.5%, 40.2, and 14.8% for REF, 30_200–1200, and 30_0.1–40, respectively.

## 4. Conclusions

In this study, the influence of share (0, 10, 20, and 30%) and fractions of particle size of waste glass (0.1–1200, 200–1200, 100–250, 63–120, 40–64, and 0.1–40 μm) on the properties of coal fly ash-based geopolymers were determined. The thermal phenomena that occurred during the geopolymerization and curing process were characterized. The consistencies of fresh mortars were examined afterward. Next, the mineralogical composition, specific surface area, water absorption, density, compressive strength, and microstructure of geopolymer composites were investigated. Finally, for all variants of the investigated samples, a fire resistance test was performed after which weight loss, residual compressive strength, and chemical composition were determined. Based on the obtained results, the following was found:

1. Thermal phenomena occurring in coal fly ash-based geopolymers closely depended on the composition of the mix. Each fraction of the particle size of waste glass additive applied in an amount of 20% by weight reduced the final setting time of the geopolymer products. Furthermore, the highest energy of the exothermic reaction was released during the geopolymerization of the sample containing the smallest particles of waste glass addition (0.1–40 μm). It is noteworthy that the sample, containing 20% of unsorted waste cullet, had the shortest final setting time of all investigated materials.
2. Decreasing the particle size of waste glass reduced the flow diameter and Novikov’s cone immersion depth of fresh geopolymer mortars. This effect was due to the increase of the specific surface area of decreasing waste glass, which showed a tendency to absorb a larger amount of alkaline solution. All fresh geopolymer mortars, containing up to 20% of waste glass, regardless of the particle size, had plastic consistency. In contrast, the addition of 30% of the waste cullet with particle sizes of 0.1–1200 µm or 200–1200 µm resulted in a change in the consistency of the mortar to liquid/thin.
3. Samples without the addition of waste glass were characterized by the largest specific surface area. Its decrease was observed with a decrease in the size of glass particles and an increase in its share in the composite. All nitrogen adsorption–desorption isotherms of geopolymers were classified as type IV isotherms with H3 hysteresis loops, indicating the presence of mesopores. Furthermore, it was found that the smaller the particles and the higher the share of the waste glass, the lower the pore volume and diameter of the pores were.
4. The water absorption of geopolymers was approximately 31%, 39%, and 43% lower for samples containing 10, 20, and 30% of waste glass with particle sizes ranging between 0.1 and 1200 μm than for reference ones. Furthermore, the addition of waste glass resulted in an increase of density by 9% (from 1.61 to 1.76 g cm^−3^), a decrease in the specific surface area by 26% (from 45.44 m^2^ g^−1^ to 33.67 m^2^ g^−1^), and a reduction of the total pore volume by 22% (from 0.1306 to 0.1013 cm^3^ g^−1^) by comparing the reference sample with geopolymers containing 30% of waste glass with particle sizes of 0.1–1200 μm simultaneously
5. Compared to the reference samples without the addition of waste glass, the compressive strength before and after the fire resistance test of the specimens containing 20% of unsorted waste glass (particle size within the range of 0.1–1200 μm) was enhanced by 135.2% and 164.7%, respectively.
6. Despite the fact that the compressive strength of the geopolymer composites increased along with the reduction of waste glass size, the use of a larger fraction of glass cullet resulted in a higher residual compressive strength determined after exposure to high temperatures. Moreover, all geopolymer samples were qualified as materials class A1_fl_ in terms of fire resistance.

The presented results are significant for the development of materials used as paving bricks, offshore structures, fireproof covers, and prefabricated elements that will characterize appropriate crucial properties such as consistency, density, water absorption, compression strength, and fire resistance while reducing the negative environmental impact. By changing the share and particle size of waste glass, the properties of fly ash-based geopolymer composites can be adapted to a certain extent to the individual requirements in the industrial sectors.

## Figures and Tables

**Table 1:** Particle size distribution for starting materials [49].

Raw Materials	D_10_	D_50_	D_90_	Mean	Span (D_90_-D_10_)/D_50_
	[µm]	[µm]	[µm]	[µm]	[µm]
Fly ash	2.3 ± 0.1	12.3 ± 1.3	37.0 ± 6.0	17.3 ± 2.5	2.8 ± 0.2
Sand	203.3 ± 13.6	416.2 ± 14.9	583.6 ± 17.2	450.1 ± 17.2	0.9 ± 0.02
0.1–1200 waste glass	112.8 ± 8.6	483.4 ± 19.7	896.7 ± 51.1	550.1 ± 18.9	1.6 ± 0.2
200–1200 waste glass	303.1 ± 1.6	584.9 ± 4.4	1123.3 ± 5.5	702.4 ± 1.6	1.4 ± 0.02
100–250 waste glass	30.4 ± 1.5	155.2 ± 0.5	248.6 ± 0.2	160.6 ± 0.6	1.4 ± 0.01
63–120 waste glass	6.3 ± 0.1	55.4 ± 1.2	118.5 ± 1.2	60.8 ± 0.9	2.0 ± 0.02
40–63 waste glass	4.9 ± 0.1	33.3 ± 0.1	63.1 ± 0.2	35.1 ± 0.1	1.7 ± 0.01
0.1–40 waste glass	3.9 ± 0.8	19.8 ± 0.3	39.2 ± 0.2	22.0 ± 0.3	1.8 ± 0.2

**Table 2:** The composition of geopolymer mixtures depending on the share and the fraction of waste glass particle size.

		Composition		Characterization of Used Waste Glass		
Sample Designation	Fly Ash [%]	Sand [%]	Waste Glass [%]	The Particle Size of Waste Glass [μm]	D_50_ of Waste Glass [μm]	Specific Surface Area [m^2^ g^−1^]
REF	90	10	-	-	-	-
10_0.1–1200	80	10	10	0.1–1200	483.4 ± 19.7	0.152
10_200–1200	80	10	10	200–1200	584.9 ± 4.4	0.048
10_100–250	80	10	10	100–250	155.2 ± 0.5	0.114
10_63–120	80	10	10	63–120	55.4 ± 1.2	0.375
10_40–63	80	10	10	40–63	33.3 ± 0.1	0.594
10_0.1–40	80	10	10	0.1–40	19.8 ± 0.3	0.693
20_0.1–1200	70	10	20	0.1–1200	483.4 ± 19.7	0.152
20_200–1200	70	10	20	200–1200	584.9 ± 4.4	0.048
20_100–250	70	10	20	100–250	155.2 ± 0.5	0.114
20_63–120	70	10	20	63–120	55.4 ± 1.2	0.375
20_40–63	70	10	20	40–63	33.3 ± 0.1	0.594
20_0.1–40	70	10	20	0.1–40	19.8 ± 0.3	0.693
30_0.1–1200	60	10	30	0.1–1200	483.4 ± 19.7	0.152
30_200–1200	60	10	30	200–1200	584.9 ± 4.4	0.048
30_100–250	60	10	30	100–250	155.2 ± 0.5	0.114
30_63–120	60	10	30	63–120	55.4 ± 1.2	0.375
30_40–63	60	10	30	40–63	33.3 ± 0.1	0.594
30_0.1–40	60	10	30	0.1–40	19.8 ± 0.3	0.693

**Table 3:** Chemical compositions of fly ash, sand, and waste glass determined using an X-ray Fluorescence Spectrometer (XRF).

Compound [%]	Fly Ash	Sand	Waste Glass
SiO_2_	52.861	89.099	68.814
Al_2_O_3_	26.561	6.589	1.598
Fe_2_O_3_	7.588	0.708	0.423
CaO	4.698	0.503	11.671
K_2_O	1.567	2.260	0.500
MgO	1.567	0.416	1.325
TiO_2_	1.370	0.141	0.083
SO_3_	1.095	0.127	0.051
MnO	0.108	0.024	0.050
P_2_O_5_	0.220	-	-
V2O5	0.089	-	-
Cr_2_O_3_	0.038	-	0.047
SrO	0.081	0.018	0.024
ZrO_2_	0.035	-	0.019
PbO	0.013	-	0.010
ZnO	0.032	0.003	0.005
NiO	0.012	-	-
SnO_2_	0.011	-	-
Ga_2_O_3_	0.006	0.005	-
Y_2_O_3_	0.012	0.002	0.002
Cs_2_O	-	0.086	-
Na_2_O	-	-	15.285
BaO	-	-	0.084

**Table 4:** Characteristic values for investigated geopolymer compositions calculated based on thermal effects recorded during the geopolymerization process of fresh geopolymer mortars, and after 48 h of curing.

Sample Designation	Maximum Temperature during the Geopolymerization Process [°C]	Time Corresponding to the Highest Temperature [s]	Difference between the Maximum Values of Thermal Effects [°C]	Final Setting Time [s]	Energy of Exothermic Reaction [J (m × K)^−1^]
REF	77.62	7797	3.19	26,580	0.66
REF without sand	77.25	7687	3.09	28,440	0.69
20_0.1–1200	76.65	7375	2.92	14,880	0.56
20_200–1200	77.00	7797	2.79	25,380	0.69
20_0.1–40	79.40	6943	6.36	24,240	2.60
30_0.1–1200	77.10	8446	2.23	56,880	0.62

**Table 5:** The specific surface area of the selected geopolymers depending on the share and the fraction of waste glass particle size.

Sample Designation	Specific Surface Area [m^2^ g^−1^]		Pore Volume [cm^3^ g^−1^]		Pore Size [nm]	
	Multi Point BET Method	Single Point BET Method	Total Pore Volume at P/P0 = 0.99	BJH Pore Volume	Average Pore Diameter	BJH Average Pore Diameter
REF	45.44	42.03	0.1306	0.1293	11.50	6.551
10_0.1–1200	44.39	40.76	0.1133	0.1120	10.21	6.544
20_0.1–1200	43.74	40.43	0.1059	0.1043	9.682	5.630
30_0.1–1200	33.26	31.13	0.09313	0.09171	11.20	7.799
30_200–1200	33.67	30.80	0.1013	0.1004	12.03	6.549
30_100–250	33.13	30.28	0.09000	0.08903	10.87	4.908
30_63–120	28.65	26.63	0.08923	0.08820	12.46	5.626
30_40–63	5.91	5.43	0.01316	0.01276	8.917	1.690
30_0.1–40	5.35	5.16	0.01141	0.01085	8.529	2.744

**Table 6:** Chemical composition of geopolymers depending on the share and the fraction of waste glass particle size before and after the fire resistance test.

Compound [%]	Before the Fire Resistance Test			After the Fire Resistance Test		
	REF	30_200–1200	30_0.1–40	REF	30_200–1200	30_0.1–40
SiO_2_	46.764	46.762	49.035	57.009	54.439	56.620
Na_2_O	21.564	27.372	23.861	16.071	16.371	20.333
Al_2_O_3_	13.553	11.653	10.576	10.877	13.329	8.462
Fe_2_O_3_	6.481	5.041	5.598	5.232	4.453	3.586
CaO	5.726	4.541	5.535	3.314	3.883	5.341
K_2_O	2.921	2.151	2.535	2.714	2.253	1.543
TiO_2_	1.104	0.838	0.925	0.957	0.783	0.590
MgO	0.717	0.843	0.649	1.007	0.846	1.376
SO_3_	0.435	0.291	0.715	2.217	3.020	1.749
P_2_O_5_	0.329	0.231	0.179	0.266	0.298	0.148
MnO	0.087	0.071	0.081	0.088	0.084	0.068
SrO	0.076	0.049	0.067	0.044	0.043	0.036
V_2_O_5_	0.067	0.053	0.073	0.066	0.096	0.046
ZrO_2_	0.035	0.026	0.031	0.029	0.027	0.024
ZnO	0.029	0.018	0.024	0.027	0.024	0.018
Cr_2_O_3_	0.023	0.028	0.039	0.021	0.018	0.030

## Data Availability

Not applicable.
